# Selective Depletion of ZAP-Binding CpG Motifs in HCV Evolution

**DOI:** 10.3390/pathogens12010043

**Published:** 2022-12-27

**Authors:** Sanket Mukherjee, Akhil Kumar, Jasmine Samal, Ekta Gupta, Perumal Vivekanandan, Manoj B. Menon

**Affiliations:** 1 Kusuma School of Biological Sciences, Indian Institute of Technology Delhi, New Delhi 110016, India; 2 Institute of Liver and Biliary Sciences, New Delhi 110070, India

**Keywords:** HCV, CpG depletion, ZAP-binding motif, pathogenesis, evolution, HCV core gene, DAA therapy

## Abstract

Hepatitis C virus (HCV) is a bloodborne pathogen that can cause chronic liver disease and hepatocellular carcinoma. The loss of CpGs from virus genomes allows escape from restriction by the host zinc-finger antiviral protein (ZAP). The evolution of HCV in the human host has not been explored in the context of CpG depletion. We analysed 2616 full-length HCV genomes from 1977 to 2021. During the four decades of evolution in humans, we found that HCV genomes have become significantly depleted in (a) CpG numbers, (b) CpG O/E ratios (i.e., relative abundance of CpGs), and (c) the number of ZAP-binding motifs. Interestingly, our data suggests that the loss of CpGs in HCV genomes over time is primarily driven by the loss of ZAP-binding motifs; thus suggesting a yet unknown role for ZAP-mediated selection pressures in HCV evolution. The HCV core gene is significantly enriched for the number of CpGs and ZAP-binding motifs. In contrast to the rest of the HCV genome, the loss of CpGs from the core gene does not appear to be driven by ZAP-mediated selection. This work highlights CpG depletion in HCV genomes during their evolution in humans and the role of ZAP-mediated selection in HCV evolution.

## 1. Introduction

Hepatitis C infection is a common cause of chronic liver disease and cirrhosis worldwide [1,2]. The current estimates of the global prevalence of hepatitis C virus (HCV) infection show that 0.7% of the global population is infected by HCV, accounting for 56.8 million cases as of January 2020 [3]. The WHO estimated that in 2019, around 290,000 people died from hepatitis C mostly from cirrhosis and hepatocellular carcinoma [4]. The Hepatitis C virus is a positive sense strand RNA virus belonging to the family *Flaviviridae*. Although there are 7 genotypes with over 65 subtypes, genotype 1 is the most prevalent, followed by genotypes 2 and 3 [5]. Other genotypes are geographically restricted [6].

The abundance of the CpG dinucleotides in viruses and their association with virus evolution and host-evasion strategies have been explored for both RNA [7,8] and DNA viruses [9,10]. The CpG content differs significantly amongst viruses [11,12]. The depletion of CpG dinucleotides in DNA viruses is attributed to several mechanisms, including the stimulation of TLR-9 immune responses and deamination of cytosines that are methylated by DNA methyl transferases. [10,11]. Although CpG dinucleotides in RNA virus genomes are not methylated, most RNA viruses infecting humans are CpG depleted [12]. A recent report identified selective binding of Zinc-finger Antiviral Protein (ZAP) to CpG enriched motifs in viral genomes leading to the restriction of virus replication [13]. Furthermore, the sequence of specific ZAP-binding motifs has also been identified [14]. The underlying mechanisms of ZAP-mediated restriction of RNA viruses is not well understood. CpG depletion in SARS CoV2 has been linked to ZAP-mediated selection pressure [7].

The majority of individuals infected with HCV progress to chronic HCV infection. Therefore, HCV has long-term interactions with the human host. Although the impact of HCV on the methylation of host genes has been studied [15], the loss of CpG dinucleotides from the HCV genomes during the last four decades of evolution in humans has not been studied. Furthermore, the role of ZAP-mediated pressures, if any, in the evolution of HCV remains unknown. The scenario for the treatment of HCV infection has changed rapidly since the introduction of Direct-Action Antivirals (DAA), which was first approved for use in 2011 for genotype 1 virus variants, after which second-generation DAAs were introduced in December 2013 [16]. Most patients on DAA regimens attained sustained virological response (SVR) [17], however, treatment failure was seen in about 5–10% [18]. Moreover, DAA-mediated SVR does not eliminate the risk of development of hepatocellular carcinoma (HCC). The availability of over 3000 complete HCV genome sequences allows us to investigate CpG depletion, ZAP-mediated selection pressures, and the effect of DAAs, if any, on virus evolution.

## 2. Materials and Methods

We retrieved 3983 complete HCV genomes available in the LANL’s HCV database (https://hcv.lanl.gov/; accessed on 28 September 2022; Last GenBank update: 1 July 2022). We used the default conservative criteria of the database to exclude sequences with too many Ns (high content of non-ACTG characters), contaminants (likely contamination with a laboratory strain), synthetic sequences, sequences containing an artifactual deletion of >100 NTs, and tiny sequences (<50 bp). We analysed 2616 sequences with information on the year of sample collection for this study (Accession numbers of sequences are listed in Supplementary Table S1).

The mono and dinucleotide frequencies were calculated as percentages from the sequence length, excluding inserts (-) and Ns from the MSA of HCV sequences. The dinucleotide O/E ratio is a normalized abundance of dinucleotides against the constituent mononucleotides, which helps with understanding whether the changes in dinucleotide frequency compared to the changes in the constituent mononucleotides. The dinucleotides O/E ratios were calculated using the formula:

(1) $${\left(O/E\right)}_{X_{p}Y}=\left[f\left(X_{p}Y\right)/f\left(X\right)f\left(Y\right)\right]\times G$$

where *f* (*X_p_Y*) = observed frequency of dinucleotide, *f* (*X*) = frequency of nucleotide X, *f* (*Y*) = frequency of nucleotide Y, *G* = Genome length.

ZAP-binding motifs (i.e., C(n_m_)G(n)CG, where m = 4/5/6/7/8) in the sequences were found using *re* module (v 2.2.1). GC content was calculated using *Biopython*’s (v 1.79) built-in method.

The multiple sequence alignment (MSA) of 2616 sequences was created using mafft v7.490 [19] in a single step. Each sequence was individually aligned to the H77 reference genome (Accession ID: NC_004102.1). A python script was used to generate mapping between pre-alignment and post-alignment reference sequence positions. The resulting MSA with a total 2616 full length sequences was used to analyse the number of sequences that lost a ZAP-binding motif from each of the ZAP-binding motif sites (i.e., n = 258 ZAP-binding motif sites present in the H77 HCV reference sequence). 

The violin plots, line plots, scatter plots, and bar plots were created using *seaborn* (v 0.11.2). The moving average plot for the number of CpGs and number of ZAP-binding motifs in the reference sequence were generated in *seaborn* (v 0.11.2), and the calculations were done using the pandas’ (v 1.3.5) rolling function with a window size of 500 bp (with window labels set as the centre of the window and no points in the window were excluded from calculations). Percentages of mononucleotides and dinucleotides were plotted along a time axis of 1-year intervals. Since multiple sequences are reported each year, a 95% confidence interval band was plotted alongside the mean values. The boxplot within the violin plots depict the lower quartile, the median, and the upper quartile. *p* < 0.01 was considered statistically significant. The Mann–Whitney U test was conducted using *scipy* (v 1.7.3) to compare the medians in the violin plots, and Pearson’s correlation coefficient was determined for the numbers of CpGs and ZAP-binding motifs using *scipy* (v 1.7.3). Bar plots were created using *seaborn* (v 0.11.2) by extracting the median values of the violin plots for obtaining the loss of CpG motifs and ZAP-binding motifs, and the number of CpGs and ZAP-binding motifs were plotted as in reference sequence. The codes used for the analysis in this study are available at the Github repository (https://github.com/iamakhilverma/hcv_seqs_analysis.git; uploaded on 7 December 2022.). The statistical calculations for the barplots, *Chi*-square tests, were performed in Graphpad. 

## 3. Results

### 3.1. Loss of CpG Content and ZAP-Binding Motifs in HCV Genome

**Figure 1:**
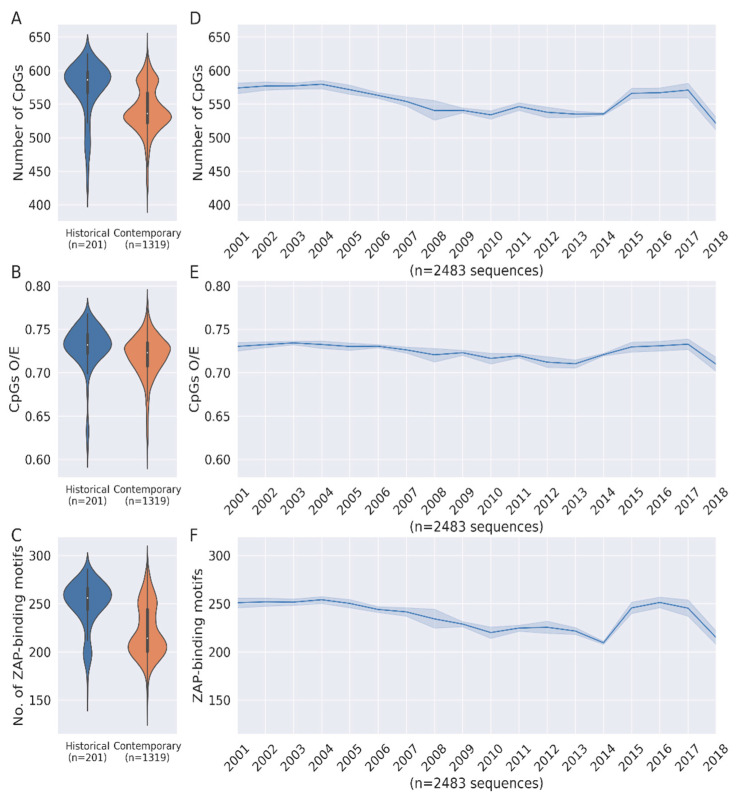
**CpGs and ZAP-binding motifs are lost during HCV genome evolution.** The violin plots depicting changes in (**A**) number of CpGs (*p* < 0.0001; Mann–Whitney *U* test), (**B**) CpGs O/E ratio (*p* < 0.0001; Mann–Whitney *U* test) and (**C**) number of ZAP-binding motifs (*p* < 0.0001; Mann–Whitney *U* test) between historical and contemporary HCV genomes. Line plots for changes in (**D**) number of CpGs, (**E**) CpGs O/E ratio and (**F**) number of ZAP-binding motifs from 2001 to 2018. The 95% confidence interval is represented by blue bands.

Full-length HCV genomes were downloaded from the HCV sequence database. We analysed a total of 2616 HCV full-length HCV genomes available from 1977 to 2022 (please see methods for details). To investigate the changes in CpG content of HCV genomes during human evolution, we analysed historical HCV sequences (i.e., all HCV sequences available from samples collected on or before 2001; n = 201 sequences) and contemporary HCV sequences (i.e., all HCV sequences available from samples collected on or after 2010; n = 1319 sequences). Interestingly, the median CpG numbers from historical HCV sequences were significantly higher than that from contemporary HCV sequences (Figure 1A; Median 586 vs. 536; *p* < 0.0001). Our data suggests that over 40 (median) CpGs were lost from HCV genomes during their four decades of evolution in humans. 

A reduction in GC% of the virus genomes can result in the reduction of CpG numbers. To investigate whether the reduction in CpG numbers in HCV genomes during their evolution in humans is merely a reflection of variation in the GC%, we estimated the relative abundance CpG dinucleotides, which is calculated as O/E (observed/expected) ratios (i.e., CpG dinucleotide content normalized to the numbers of the constituent C and G mononucleotides). If the loss of Cs and Gs from the genome is resulting in the loss of CpG dinucleotides from HCV genomes, the O/E ratios of the historical and contemporary HCV sequences will be comparable. On the contrary, decreasing O/E ratios of HCV genomes over time suggest that the loss of CpGs is independent of the GC% (Figure 1B). The validity of this finding is further strengthened by the fact that there is indeed a significant reduction in the GC content of HCV genomes during this period (Supplementary Figure S1). We then wanted to investigate the CpG content of HCV genotypes. However, the availability of very few sequences (i.e., less than 25 sequences) from before 2001 precluded this analysis of HCV genotypes with the exception of genotype 1. Loss of CpGs, CpG O/E ratios, and ZAP-binding motifs with time were observed for HCV genotype 1 (Supplementary Figure S2). The lack of an adequate number of sequences from other HCV genotypes/subtypes did not allow us to elucidate genotype/subtype-specific differences in CpG content and ZAP-binding motifs, if any, across HCV genomes. Although there is no genotype-specific selection bias in the sequences analysed in our study, we cannot rule out a role for the differences in the distribution of HCV genotypes over time and its potential impact on our findings.

CpG-rich regions can be targets for ZAP-mediated virus restriction. ZAP was recently shown to bind to diverse sequence motifs with a terminal CpG dinucleotide (i.e., C(n4-8)G(n)CG), where n = a/c/g/t) [14]. ZAP is known to inhibit viral replication by binding to CpG-rich regions of the viral genome [13]. Contemporary HCV sequences had significantly lower numbers of ZAP-binding motifs as compared to historical HCV sequences (Figure 1C). Together, our data suggest that during evolution in humans, HCV genomes have lost CpGs (both absolute numbers and relative abundance) as well as ZAP-binding motifs. HCV is one of the few RNA viruses that has a long-term relationship with the host. Pronounced depletion of CpG content and ZAP-binding motifs in the HCV genome during its evolution in the human host may represent a strategy for evasion of host innate immune responses. 

We also plotted CpG numbers, CpG O/E ratios, and the number of ZAP-binding motifs in HCV genomes year-wise from 2001 to 2018 (Figure 1D–F). The HCV sequences from samples collected before 2001 and after 2018 were excluded since less than 10 sequences were available for a given year. The line plots indicate a steady decline of CpG numbers, CpG O/E ratios, and the number of ZAP-binding motifs from 2001 to 2013; this is followed by an increase between 2013 and 2015. There is again a sharp dip of all three parameters from 2016 onwards. Overall, the line plots show a dip in CpG numbers, CpG O/E ratios, and the number of ZAP-binding motifs in HCV genomes from 2001 to 2018 (Figure 1D–F).

### 3.2. Correlation between Loss of ZAP Binding Motif and CpG Loss

**Figure 2:**
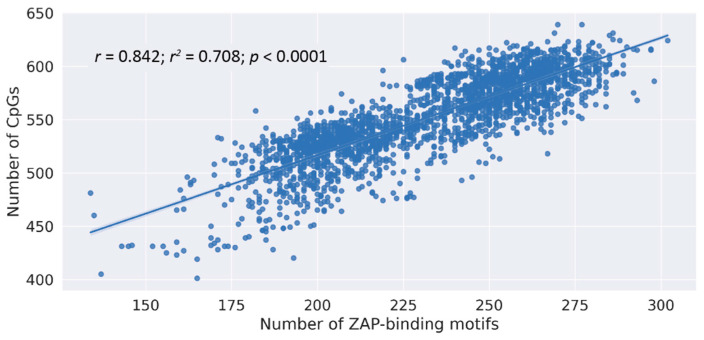
**Correlation between the number of CpGs and the number of ZAP-binding in HCV genomes**. The correlation plot between the number of CpGs and the number of ZAP-binding motifs (r = 0.84, r^2^ = 0.71, *p* < 0.0001; Pearson’s correlation coefficient with two-tailed *p* value test).

ZAP-binding motifs contain a terminal CpG. Nonetheless, it is not known whether the number of CpGs in virus genomes correlates with the number of ZAP-binding motifs. Since ZAP-binding motifs and CpGs are both lost from HCV genomes with time, we investigated the correlation, if any, between CpG numbers and the number of ZAP-binding motifs. We found a strong correlation between CpG numbers and the number of ZAP-binding motifs in the HCV genome (Figure 2; r = 0.84; *p* < 0.0001; Pearson’s r statistical calculations). Our results indicate that the CpG numbers in the HCV genome may serve as a surrogate for the number of ZAP-binding motifs. 

### 3.3. CpG Depeletion in HCV Genomes Are Primarily Driven by the Loss of ZAP-Binding Motifs

**Figure 3:**
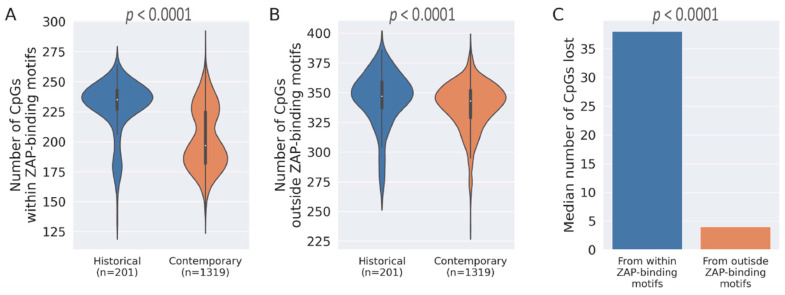
**CpG depletion in HCV genomes is primarily driven by the loss of CpGs from ZAP-binding motifs.** (**A**) Violin plot for the loss of CpG motifs within ZAP-binding motifs in contemporary sequences compared to the historical sequences (*p* < 0.0001; Mann–Whitney *U* test). (**B**) Violin plot for the loss of CpG motifs outside ZAP-binding motifs in contemporary sequences compared to the historical sequences (*p* < 0.0001; Mann–Whitney *U* test). (**C**) Median loss of CpGs within ZAP-binding motifs and outside ZAP-binding motifs (*p* < 0.0001; *Chi*-square test).

The analysis of CpG dinucleotides in historical and contemporary HCV sequences reveals that over 40 CpGs are lost during evolution in the human host (Figure 1A). To understand if ZAP-mediated selection pressures are major drivers of CpG depletion in HCV genomes, we assessed the median numbers and the proportion of CpGs lost from within ZAP-binding motifs and outside ZAP-binding motifs. The majority of the CpGs lost [38 of 50 CpGs lost (76%) over time were from within ZAP-binding motifs] were lost due to the loss of the CpGs within ZAP-binding motifs (Figure 3A,B), suggesting that ZAP-mediated selection pressures may be the major drivers of CpG depletion during the evolution of HCV genomes in the human host. Our findings clearly support evolutionary or survival advantages for the loss of CpGs from ZAP-binding motifs in the HCV genome as opposed to the loss of CpGs that lie outside ZAP-binding motifs (Figure 3C).

### 3.4. Selective Conservation of Specific ZAP-Binding Motifs during HCV Evolution

**Figure 4:**
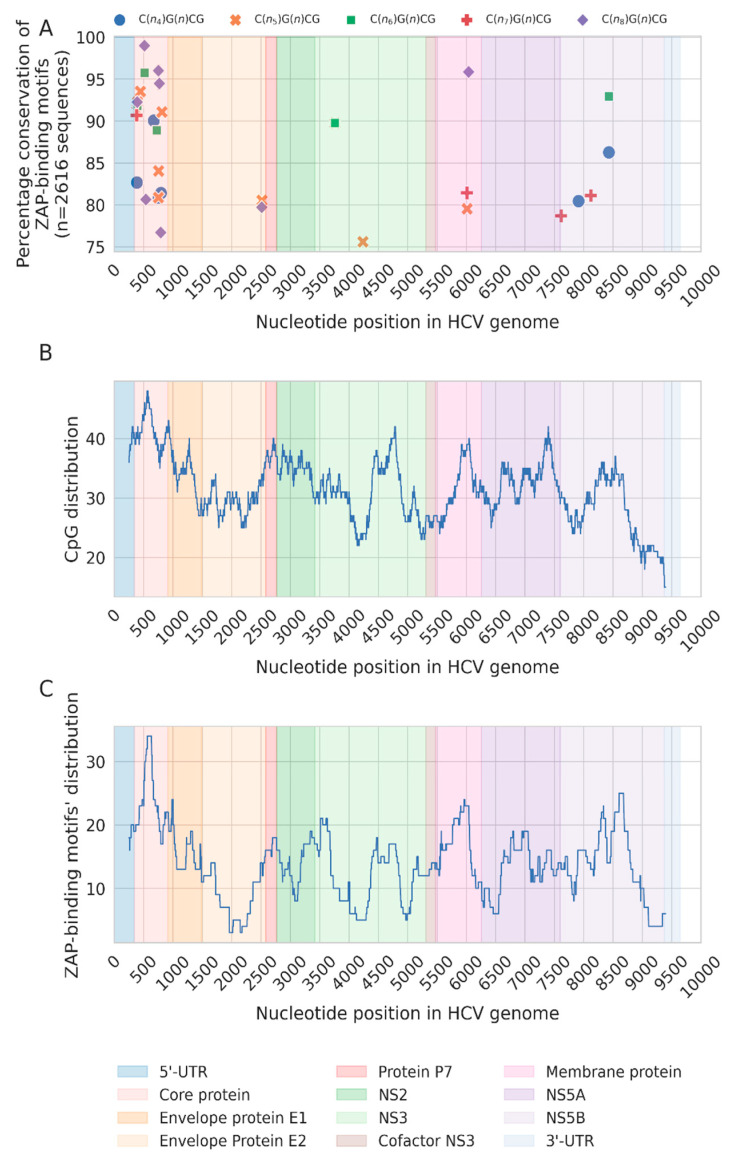
**Conservation of ZAP-binding motif and the distribution of CpG and ZAP-binding motifs in HCV genomes** (**A**). The conservation plot of ZAP-binding motifs across the entire length of the HCV genome. Different ZAP-binding motif consensus identified are indicated. A map of all the ZAP-binding motifs and those conserved in ≥75% of the HCV sequences is provided in Supplementary Figure S3. (**B**,**C**). Moving average plot of ZAP-binding motifs (**B**) and CpG motifs (**C**)with a sliding window size of 500 nucleotides are shown. The genomic coordinates are indicated with legend for the HCV genes and genomic elements.

Temporal loss of ZAP-binding motifs from HCV genomes is evident in our results (Figure 1E). However, to visualize the loss of ZAP-binding motifs at specific genome locations, data on the conservation of each ZAP-binding motif along the length of the whole HCV genomes were plotted. These data from all the 2616 sequences were mapped to the reference HCV genomic sequence H77 (Accession number: NC_004102) (Figure 4A). Of note, only about 5% of the 258 predicted ZAP-binding sites in the HCV reference genome were conserved in ≥90% HCV genomes analysed (Figure 4A). Interestingly, out of the 30 ZAP-binding sites conserved in >75% of the genomes, 18 were present within the first 1000 nucleotides of the genome showing a clear enrichment at the core protein region of the genome (Figure 4A). The moving average plot of CpGs and ZAP-binding motifs across the genome also revealed a similar picture, where the gene encoding the HCV core protein (342-914 nucleotide positions) is enriched for CpGs and ZAP-binding motifs (Figure 4B,C).

Despite a good correlation between the number of CpGs and ZAP-binding motifs in HCV genomes (Figure 2), specific genomic regions appear to be exceptions. For example, the genes coding for E1 and NS3 proteins appear to have high CpG numbers but relatively fewer ZAP-motifs (Figure 4B,C). The genes encoding the HCV core protein, membrane protein, and NS5B are enriched for ZAP-binding motifs (Figure 4C). Interestingly, the conservation of ZAP-binding motif is much more pronounced in the HCV Core gene compared to those encoding the membrane protein or NS5B (Figure 4A). 

### 3.5. Enhanced Loss of CpGs from the HCV Core Gene from Outside ZAP-Binding Motifs 

**Figure 5:**
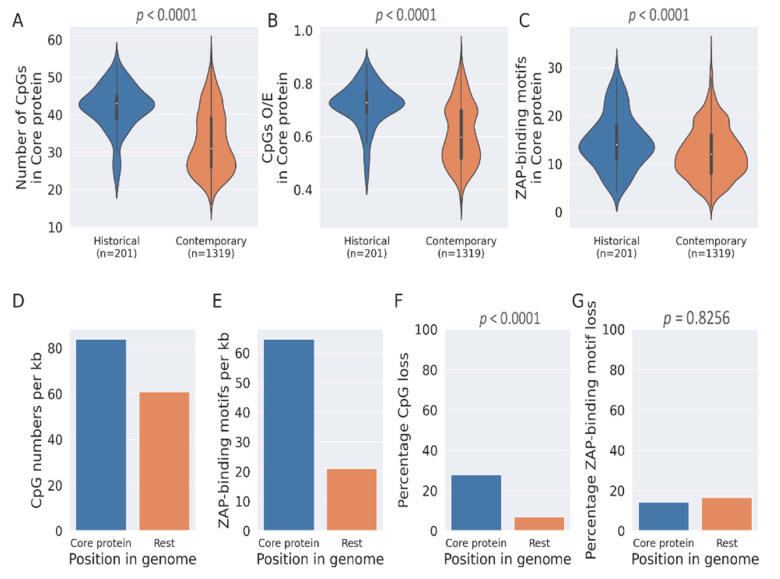
**The HCV core gene preferentially loses CpGs that are outside the ZAP-binding motif.** (**A**–**C**). The changes in (**A**) number of CpGs (*p* < 0.0001; Mann–Whitney *U* test), (**B**) CpGs O/E (*p* < 0.0001; Mann–Whitney *U* test) and (**C**) number of ZAP-binding motifs (*p* < 0.0001; Mann–Whitney *U* test) in the HCV core protein encoding region of the genome. (**D**). Enrichment of CpGs in core protein region. (**E**). Enrichment of ZAP-binding motifs in the core protein region. (**F**) Relative loss of CpG in the core protein region compared with the rest of the genome (*p* < 0.0001; *Chi*-square test). (**G**)**.** Relative loss of ZAP-binding motifs in the core protein region compared with the rest of the genome (*p* = 0.8256; *Chi*-square test).

The high CpG numbers and ZAP-binding motifs in the HCV core gene may also imply that genes encoding key structural proteins of a virus are more resistant to mutations and/or loss of CpGs. We then investigated the high numbers of CpGs and ZAP-binding motifs in the HCV core gene. Previous reports on the HCV core gene suggest a higher degree of conservation to maintain the structural integrity of the virus [20,21]. We therefore investigated the loss of CpGs and ZAP-binding motifs in this region as compared to the rest of the genome. There were significant decreases in CpG numbers, CpG O/E ratios, and number of ZAP-binding motifs in the core-protein encoding region (nucleotide positions 342–914) of the viral genome (Figure 5A–C). Both CpG dinucleotides and ZAP-binding motifs are enriched in the core protein region as compared to the rest of the genome (Figure 5D,E). When we plotted the proportion of CpGs lost (median) from the core gene and the rest of the genome, the proportion of CpGs lost from the core gene was almost four-fold higher than that from the rest of the HCV genome (Figure 5F). Of note, the proportion of ZAP-binding motifs lost from the core gene is marginally lower than that from the rest of the genome (Figure 5G). Interestingly, although the proportion of total CpGs lost from the HCV core gene is much higher than that from the rest of the genome, the loss of ZAP-binding motifs is not the primary driver of CpG loss within the core gene; this is in contrast to the finding at the whole genome level. This finding explains at least in part the conservation ZAP-binding motifs, a phenomenon that was unique to the HCV core gene (Figure 4A). Nonetheless, the mechanisms underlying the enhanced CpG loss from outside the ZAP-binding motifs within the HCV core gene merits investigation.

## 4. Discussion

High mutation rates, recombination, and mutations in the virus polymerase have been associated with the high genetic diversity of the HCV genome [22]. As a result, HCV genotypes may have up to 30% genetic diversity at specific genomic regions [23]. In addition to subtypes within a genotype, HCV quasispecies or viral variants with an infected host adds to its diversity [24]. 

Studies on CpG depletion in other RNA viruses have provided interesting insights on virus evolution, pathogenesis, and adaptation to the host [25,26,27,28]. Therefore, the evolution of CpGs HCV in humans over the last four decades represents an interesting but yet unexplored opportunity. 

We analysed a total of 2616 HCV genomes from 1977 to 2021 and found a significant reduction in CpG numbers, CpG O/E ratios, and ZAP-binding motifs over time. Contemporary HCV genomes have significantly reduced CpG content and ZAP-binding motifs as compared to historical HCV sequences. These findings suggest a role for CpG depletion in shaping the evolution of HCV. Previous studies have shown that CpG depletion in virus genomes is pronounced during host adaptations [7,27]. In addition, CpG content remains stable for well-adapted human viruses such as influenza B virus [27]. Our findings indicate that CpG content for HCV genomes still appears to be evolving, suggesting ongoing adaptations to the human host. This is consistent with a report that suggests that the most common ancestor of HCV (subtype 1b) infections in humans may date back to early 1900s [29].

The trend of declining CpG numbers, CpG O/E ratios, and the number of ZAP-binding motifs in the HCV genome over time was briefly reversed during the period 2013–2015 (Figure 1D–F). An increase in CpG content and ZAP-binding motifs in HCV genomes is evident from 2013–2015. Interestingly, this period overlaps with the timeline for the approval of combination DAA therapy [30]. The introduction of antiviral drugs may limit the genetic diversity of viruses in the host, as only a small subset of the virus population with resistant mutations are able to survive. This genetic bottleneck may also lead to the emergence of new drug-resistant variants [31]. We speculate that evolutionary constraints associated with the introduction of combination DAA therapy for HCV may have impacted the evolution of CpGs and ZAP-binding motifs from 2013 to 2015. Previous reports indicate that Ribavarin (anti-HCV agent) leads to the accumulation of mutations at specific genomic locations [32]. Furthermore, some of the DAA anti-HCV drugs target the HCV RNA dependent polymerase [30], which may directly impact the type of mutations occurring in the HCV genome 

Apart from ZAP-mediated selection pressures, other selection pressures including TLR7-mediated immune selection pressures [7], host-specific selection pressures [12], and tissue-specific selection pressures [26] may be associated with the depletion of CpGs in RNA viruses. Therefore, the number of CpGs may not necessarily correlate with the number of ZAP-binding motifs for a given RNA virus. Nonetheless, we found a good correlation between the CpG numbers and the number of ZAP-binding motifs in HCV genomes (Figure 2). This finding suggests that CpG numbers in HCV genomes may be surrogates for the number of ZAP-binding motifs. 

The role of ZAP-mediated selection pressures in shaping RNA virus evolution has not been well studied. In SARS-CoV-2, the depletion of CpGs has been primarily attributed to pressures acting outside the ZAP-binding motifs. Our finding on the role of ZAP-mediated selection pressures as a major driver of HCV evolution (Figure 3) highlights that the CpG depletion in RNA viruses infecting humans is due to fundamental differences in evolutionary pressures. The underlying reasons for contrasting roles of ZAP-mediated selection among human viruses remain elusive. Liver is one of the human tissues where ZAP is highly expressed (Tissue atlas) [33]. A potential role for tissue-specific expression of ZAP and the necessary co-factors for ZAP-mediated restriction merits further investigation.

Among the HCV genes, the HCV core gene is enriched for both CpGs and ZAP-binding motifs. Although ZAP-binding motifs in the HCV genome are depleted with time due to selection pressures, ZAP-binding motifs within the HCV core gene appear to be well conserved (Figure 4). The HCV core protein is a basic protein that interacts with HCV RNA, and oligomerizes and facilitates virus assembly [34]. In addition, the HCV core protein is a nucleic acid chaperone [35]. Mutations and deletions in the N-terminus of the HCV core has been shown to impact virus assembly [36]. We have not identified the specific reasons for the conservation of ZAP-binding motifs in the HCV core gene. Nonetheless, the selective conservation of ZAP-binding motifs in specific genes in virus genomes may indicate the existence of yet unknown constraints that minimize the loss of CpGs/ZAP-binding motifs. Importantly, this finding also suggests that the benefits of retaining CpGs/ZAP-binding motifs over the survival/replication advantages are associated with escaping ZAP-mediated restriction in the host. We also found that the loss of CpGs within the HCV core gene occurs primarily outside ZAP-binding motifs (Figure 5), suggesting the existence of gene-specific differences in selection pressures. 

## 5. Conclusions

In conclusion, here we identify a role for CpG depletion in shaping HCV evolution in the human host. Our results also suggest that ZAP-mediated selection pressures are the major drivers of CpG depletion in the HCV genome. The conservation of ZAP-binding motifs in the HCV genome is unique to the HCV core gene, where CpG depletion is primarily driven by selection pressures that are independent of ZAP-mediated restriction. This work highlights the underlying mechanisms of CpG depletion in HCV genomes in humans and sheds light on the contrasting role of different selection pressures at specific genomic locations within a virus genome.

## Data Availability

All data are available on request.

## Supplementary Materials

The following supporting information can be downloaded at: https://www.mdpi.com/article/10.3390/pathogens12010043/s1, Figure S1: GC content of HCV genomes over time; Figure S2: Analysis of HCV genotype 1; Figure S3. Analysis of conservation of ZAP-binding motifs in the HCV reference sequence (NC 004102); Table S1: Accession Numbers of HCV sequences.

## References

[1] National Institutes of Health (2002). National Institutes of Health Consensus Development Conference Statement: Management of Hepatitis C 2002 (June 10–12, 2002). Gastroenterology.

[2] Sievert W., Altraif I., Razavi H.A., Abdo A., Ahmed E.A., Alomair A., Amarapurkar D., Chen C.H., Dou X., el Khayat H. (2011). A Systematic Review of Hepatitis C Virus Epidemiology in Asia, Australia and Egypt. Liver Int..

[3] Blach S., Terrault N.A., Tacke F., Gamkrelidze I., Craxi A., Tanaka J., Waked I., Dore G.J., Abbas Z., Abdallah A.R. (2022). Global Change in Hepatitis C Virus Prevalence and Cascade of Care between 2015 and 2020: A Modelling Study. Lancet Gastroenterol. Hepatol..

[4] Hepatitis C. https://www.who.int/news-room/fact-sheets/detail/hepatitis-C.

[5] Smith D.B., Bukh J., Kuiken C., Muerhoff A.S., Rice C.M., Stapleton J.T., Simmonds P. (2014). Expanded Classification of Hepatitis C Virus into 7 Genotypes and 67 Subtypes: Updated Criteria and Genotype Assignment Web Resource. Hepatology.

[6] Simmonds P., McOmish F., Yap P.L., Dow B.C., Follett E.A.C., Seed C., Keller A.J., Cobain T.J., Krusius T., Kolho E. (1994). Geographical Distribution of Hepatitis C Virus Genotypes in Blood Donors: An International Collaborative Survey. J. Clin. Microbiol..

[7] Kumar A., Goyal N., Saranathan N., Dhamija S., Saraswat S., Menon M.B., Vivekanandan P. (2022). The Slowing Rate of CpG Depletion in SARS-CoV-2 Genomes Is Consistent with Adaptations to the Human Host. Mol. Biol. Evol..

[8] Wasson M.K., Borkakoti J., Kumar A., Biswas B., Vivekanandan P. (2017). The CpG Dinucleotide Content of the HIV-1 Envelope Gene May Predict Disease Progression. Sci. Rep..

[9] Upadhyay M., Vivekanandan P. (2015). Depletion of CpG Dinucleotides in Papillomaviruses and Polyomaviruses: A Role for Divergent Evolutionary Pressures. PLoS ONE.

[10] Upadhyay M., Samal J., Kandpal M., Vasaikar S., Biswas B., Gomes J., Vivekanandan P. (2013). CpG Dinucleotide Frequencies Reveal the Role of Host Methylation Capabilities in Parvovirus Evolution. J. Virol..

[11] Hoelzer K., Shackelton L.A., Parrish C.R. (2008). Presence and Role of Cytosine Methylation in DNA Viruses of Animals. Nucleic Acids Res..

[12] Cheng X., Virk N., Chen W., Ji S., Ji S., Sun Y., Wu X. (2013). CpG Usage in RNA Viruses: Data and Hypotheses. PLoS ONE.

[13] Takata M.A., Gonçalves-Carneiro D., Zang T.M., Soll S.J., York A., Blanco-Melo D., Bieniasz P.D. (2017). CG Dinucleotide Suppression Enables Antiviral Defence Targeting Non-Self RNA. Nature.

[14] Luo X., Wang X., Gao Y., Zhu J., Liu S., Gao G., Gao P. (2020). Molecular Mechanism of RNA Recognition by Zinc-Finger Antiviral Protein. Cell Rep..

[15] Shen J., Wang S., Zhang Y.J., Wu H.C., Kibriya M.G., Jasmine F., Ahsan H., Wu D.P.H., Siegel A.B., Remotti H. (2013). Exploring Genome-Wide DNA Methylation Profiles Altered in Hepatocellular Carcinoma Using Infinium HumanMethylation 450 BeadChips. Epigenetics.

[16] Bryan-Marrugo O.L., Ramos-Jiménez J., Barrera-Saldaña H., Rojas-Martínez A., Vidaltamayo R., Rivas-Estilla A.M. (2015). History and Progress of Antiviral Drugs: From Acyclovir to Direct-Acting Antiviral Agents (DAAs) for Hepatitis C. Med. Univ..

[17] Ferrarese A., Germani G., Gambato M., Russo F.P., Senzolo M., Zanetto A., Shalaby S., Cillo U., Zanus G., Angeli P. (2018). Hepatitis C Virus Related Cirrhosis Decreased as Indication to Liver Transplantation since the Introduction of Direct-Acting Antivirals: A Single-Center Study. World J. Gastroenterol..

[18] Zeuzem S., Mizokami M., Pianko S., Mangia A., Han K.H., Martin R., Svarovskaia E., Dvory-Sobol H., Doehle B., Hedskog C. (2017). NS5A Resistance-Associated Substitutions in Patients with Genotype 1 Hepatitis C Virus: Prevalence and Effect on Treatment Outcome. J. Hepatol..

[19] Katoh K., Standley D.M. (2013). MAFFT Multiple Sequence Alignment Software Version 7: Improvements in Performance and Usability. Mol. Biol. Evol..

[20] Ray Ayb R.B., Ray R. (2001). Hepatitis C Virus Core Protein: Intriguing Properties and Functional Relevance. FEMS Microbiol. Lett..

[21] Kunkel M., Lorinczi M., Rijnbrand R., Lemon S.M., Watowich S.J. (2001). Self-Assembly of Nucleocapsid-Like Particles from Recombinant Hepatitis C Virus Core Protein. J. Virol..

[22] Preciado M.V., Valva P., Escobar-Gutierrez A., Rahal P., Ruiz-Tovar K., Yamasaki L., Vazquez-Chacon C., Martinez-Guarneros A., Carpio-Pedroza J.C., Fonseca-Coronado S. (2014). Hepatitis C Virus Molecular Evolution: Transmission, Disease Progression and Antiviral Therapy. World J. Gastroenterol..

[23] Simmonds P., Holmes E.C., Cha T.A., Chan S.W., McOmish F., Irvine B., Beall E., Yap P.L., Kolberg J., Urdea M.S. (1993). Classification of Hepatitis C Virus into Six Major Genotypes and a Series of Subtypes by Phylogenetic Analysis of the NS-5 Region. J. Gen. Virol..

[24] Echeverría N., Moratorio G., Cristina J., Moreno P. (2015). Hepatitis C Virus Genetic Variability and Evolution. World J. Hepatol..

[25] Alinejad-Rokny H., Anwar F., Waters S.A., Davenport M.P., Ebrahimi D. (2016). Source of CpG Depletion in the HIV-1 Genome. Mol. Biol. Evol..

[26] Sankar S., Borkakoti J., Ramamurthy M., Nandagopal B., Vivekanandan P., Gopalan S. (2018). Identification of Tell-Tale Patterns in the 3′ Non-Coding Region of Hantaviruses That Distinguish HCPS-Causing Hantaviruses from HFRS-Causing Hantaviruses. Emerg. Microbes Infect..

[27] Greenbaum B.D., Levine A.J., Bhanot G., Rabadan R. (2008). Patterns of Evolution and Host Gene Mimicry in Influenza and Other RNA Viruses. PLoS Pathog..

[28] Xia X. (2020). Extreme Genomic CpG Deficiency in SARS-CoV-2 and Evasion of Host Antiviral Defense. Mol. Biol. Evol..

[29] Gray R.R., Tanaka Y., Takebe Y., Magiorkinis G., Buskell Z., Seeff L., Alter H.J., Pybus O.G. (2013). Evolutionary Analysis of Hepatitis C Virus Gene Sequences from 1953. Philos. Trans. R. Soc. B Biol. Sci..

[30] Geddawy A., Ibrahim Y.F., Elbahie N.M., Ibrahim M.A. (2017). Direct Acting Anti-Hepatitis C Virus Drugs: Clinical Pharmacology and Future Direction. J. Transl. Int. Med..

[31] Kitrinos K.M., Nelson J.A.E., Resch W., Swanstrom R. (2005). Effect of a Protease Inhibitor-Induced Genetic Bottleneck on Human Immunodeficiency Virus Type 1 Env Gene Populations. J. Virol..

[32] Contreras A.M., Hiasa Y., He W., Terella A., Schmidt E.V., Chung R.T. (2002). Viral RNA Mutations Are Region Specific and Increased by Ribavirin in a Full-Length Hepatitis C Virus Replication System. J. Virol..

[33] Uhlén M., Fagerberg L., Hallström B.M., Lindskog C., Oksvold P., Mardinoglu A., Sivertsson Å., Kampf C., Sjöstedt E., Asplund A. (2015). Proteomics. Tissue-Based Map of the Human Proteome. Science.

[34] Santolini E., Migliaccio G., la Monica N. (1994). Biosynthesis and Biochemical Properties of the Hepatitis C Virus Core Protein. J. Virol..

[35] Cristofari G., Ivanyi-Nagy R., Gabus C., Boulant S., Lavergne J.P., Penin F., Darlix J.L. (2004). The Hepatitis C Virus Core Protein Is a Potent Nucleic Acid Chaperone That Directs Dimerization of the Viral (+) Strand RNA in Vitro. Nucleic Acids Res..

[36] Klein K.C., Dellos S.R., Lingappa J.R. (2005). Identification of Residues in the Hepatitis C Virus Core Protein That Are Critical for Capsid Assembly in a Cell-Free System. J. Virol..

