# Supplementary material for: Selective Depletion of ZAP-Binding CpG Motifs in HCV Evolution
Source: Pathogens. 2022 Dec 27;12(1):43. doi: 10.3390/pathogens12010043 (PMC9866289; doi:10.3390/pathogens12010043)
Supplement: Supplementary file 1 [file pathogens-12-00043-s001.zip › Supplementary Figures _Final.pdf]

Supplementary Material

Supplementary Figure S1

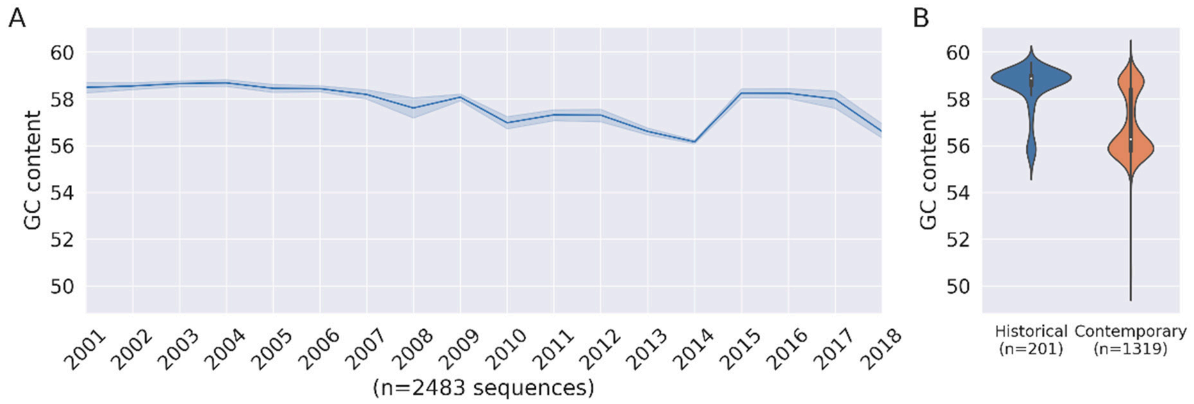

Figure S1: GC content of HCV genomes over time: **A**.Line plot showing the GC content of HCV genomes from samples collected during 2001 to 2018, **B.** Violin plots with GC content of historical and comtemporary sequences ($p < 0.0001$).

Supplementary Figure S2

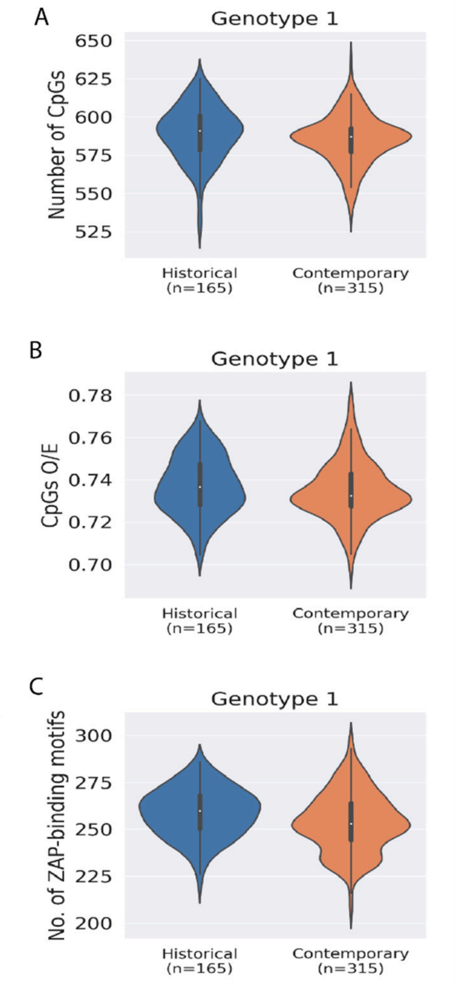

Figure S2: Analysis of HCV genotype 1: Violin plots comparing **A.** CpG numbers (p = 0.004), **B.** CpG O/E (p = 0.04), **C.** Number of ZAP binding motifs (p = 0.0002) between historical sequences to contemporary from HCV genotype 1.

Supplementary Figure S3

1 GCCAGCCCCC TGATGGGGGC GACACTCCAC CATGAATCAC TCCCCTGTGA GGAACTACTG
61 TCTTCACGCA GAAAGCGTCT AGCCATGGCG TTAGTATGAG TGTCGTGCAG CCTCCAGGAC
121 CCCCCCTCCC GGGAGAGCCA TAGTGGTCTG CGGAACCGGT GAGTACACCG GAATTGCCAG
181 GACGACCGGG TCCTTTCTTG GATAAACCCG CTCAATGCCT GGAGATTTGG GCGTGCCCCC
241 GCAAGACTGC TAGCCGAGTA GTGTTGGGTC GCGAAAGGCC TTGTGGTACT GCCTGATAGG
301 GTGCTTGCGA GTGCCCCGGG AGGTCTCGTA GACCGTGCAC CATGAGCACG AATCCTAAAC
361 CTCAAAGAAA AACCAAACGT AA**CACCAACC** **GTCGCCCACA** **GGACG**TCAAG TTCCCGGGTG
421 GCGGTCAGAT CGTTGGTGGA GTTTA**CTTGT** **TGCCG**CGCAG GGGCCCTAGA TTGGGTGTGC
481 GCGCGACGAG GAAGACTTCC GAGCGGTCGC AAC**CTCGAGG** **TAGACG**TCAG CCTATCC**CCA**
541 **AGGCACGTCG** GCCCGAGGGC AGGACCTGGG CTCAGCCCGG GTACCCTTGG CCCCTCTATG
601 GCAATGAGGG TTGCGGGTGG GCGGGATGGC TCCTGTCTCC CCGTGGCTCT CGGCCTAGCT
661 GGGGCCCCAC AGA**CCCCCGG** **CG**TAGGTCGC GCAATTTGGG TAAGGTCATC GATACCCTTA
721 CGTG**CGGCTT** **CGCCG**ACCTC ATGGGGTACA TA**CCGCTCGT** **CGGCG**CC**CCT** **CTTGGAGGCG**
781 CTGCCAGGGC **CCTGGCGCAT** **GGCG**TCCGGG TTCTGGAAGA CGGCGTGAAC TATGCAACAG
841 GGAACCTTCC TGGTTGCTCT TTCTCTATCT TCCTTCTGGC CCTGCTCTCT TGCCTGACTG
901 TGCCCGCTTC AGCCTACCAA GTGCGCAATT CCTCGGGGCT TTACCATGTC ACCAATGATT
961 GCCCTAACTC GAGTATTGTG TACGAGGCGG CCGATGCCAT CCTGCACACT CCGGGGTGTG
1021 TCCCTTGCGT TCGCGAGGGT AACGCCTCGA GGTGTTGGGT GGCGGTGACC CCCACGGTGG
1081 CCACCAGGGA CGGCAAACTC CCCACAACGC AGCTTCGACG TCATATCGAT CTGCTTGTCG
1141 GGAGCGCCAC CCTCTGCTCG GCCCTCTACG TGGGGGACCT GTGCGGGTCT GTCTTTCTTG
1201 TTGGTCAACT GTTTACCTTC TCTCCCAGGC GCCACTGGAC GACGCAAGAC TGCAATTGTT
1261 CTATCTATCC CGGCCATATA ACGGGTCATC GCATGGCATG GGATATGATG ATGAACTGGT
1321 CCCCTACGGC AGCGTTGGTG GTAGCTCAGC TGCTCCGGAT CCCACAAGCC ATCATGGACA
1381 TGATCGCTGG TGCTCACTGG GGAGTCCTGG CGGGCATAGC GTATTTCTCC ATGGTGGGGA
1441 ACTGGGCGAA GGTCCTGGTA GTGCTGCTGC TATTTGCCGG CGTCGACGCG GAAACCCACG
1501 TCACCGGGGG AAGTGCCGGC CGCACCACGG CTGGGCTTGT TGGTCTCCTT ACACCAGGCG
1561 CCAAGCAGAA CATCCAACTG ATCAACACCA ACGGCAGTTG GCACATCAAT AGCACGGCCT
1621 TGAACTGCAA TGAAAGCCTT AACACCGGCT GGTTAGCAGG GCTCTTCTAT CAGCACAAAT
1681 TCAACTCTTC AGGCTGTCCT GAGAGGTTGG CCAGCTGCCG ACGCCTTACC GATTTTGCCC
1741 AGGGCTGGGG TCCTATCAGT TATGCCAACG GAAGCGGCCT CGACGAACGC CCCTACTGCT
1801 GGCACTACCC TCCAAGACCT TGTGGCATTG TGCCCGCAAA GAGCGTGTGT GGCCCGGTAT
1861 ATTGCTTCAC TCCCAGCCCC GTGGTGGTGG GAACGACCGA CAGGTCGGGC GCGCCTACCT
1921 ACAGCTGGGG TGCAAATGAT ACGGATGTCT TCGTCCTTAA CAACACCAGG CCACCGCTGG
1981 GCAATTGGTT CGGTTGTACC TGGATGAACT CAACTGGATT CACCAAAGTG TGCGGAGCGC
2041 CCCCTTGTGT CATCGGAGGG GTGGGCAACA ACACCTTGCT CTGCCCCACT GATTGTTTCC
2101 GCAAGCATCC GGAAGCCACA TACTCTCGGT GCGGCTCCGG TCCCTGGATT ACACCCAGGT
2161 GCATGGTCGA CTACCCGTAT AGGCTTTGGC ACTATCCTTG TACCATCAAT TACACCATAT
2221 TCAAAGTCAG GATGTACGTG GGAGGGGTCG AGCACAGGCT GGAAGCGGCC TGCAACTGGA
2281 CGCGGGGCGA ACGCTGTGAT CTGGAAGACA GGGACAGGTC CGAGCTCAGC CCATTGCTGC
2341 TGTCCACCAC ACAGTGGCAG GTCCTTCCGT GTTCTTTCAC GACCCTGCCA GCCTTGTCCA
2401 CCGGCCTCAT CCACCTCCAC CAGAACATTG TGGACGTGCA GTACTTGTAC GGGGTAGGGT
2461 CAAGCATCGC GTCCTGGGCC ATTAAGTGGG AGTACGTCGT TCTCCTGTTC CTC**CTGCTTG**
2521 **CAGACG**CGCG CGTCTGCTCC TGCTTGTGGA TGATGTTACT CATATCCCAA GCGGAGGCGG
2581 CTTTGGAGAA CCTCGTAATA CTCAATGCAG CATCCCTGGC CGGGACGCAC GGTCTTGTGT
2641 CCTTCCTCGT GTTCTTCTGC TTTGCGTGGT ATCTGAAGGG TAGGTGGGTG CCCGGAGCGG
2701 TCTACGCCTT CTACGGGATG TGGCCTCTCC TCCTGCTCCT GCTGGCGTTG CCTCAGCGGG
2761 CATACGCACT GGACACGGAG GTGGCCGCGT CGTGTGGCGG CGTTGTTCTT GTCGGGTTAA
2821 TGGCGCTGAC TCTGTCGCCA TATTACAAGC GCTACATCAG CTGGTGCATG TGGTGGCTTC
2881 AGTATTTTCT GACCAGAGTA GAAGCGCAAC TGCACGTGTG GGTTCCCCCC CTCAACGTCC
2941 GGGGGGGGCG CGATGCCGTC ATCTTACTCA TGTGTGTTGT ACACCCGACT CTGGTATTTG
3001 ACATCACCAA ACTACTCCTG GCCATCTTCG GACCCCTTTG GATTCTTCAA GCCAGTTTGC
3061 TTAAAGTCCC CTACTTCGTG CGCGTTCAAG GCCTTCTCCG GATCTGCGCG CTAGCGCGGA
3121 AGATAGCCGG AGGTCATTAC GTGCAAATGG CCATCATCAA GTTAGGGGCG CTTACTGGCA
3181 CCTATGTGTA TAACCATCTC ACCCCTCTTC GAGACTGGGC GCACAACGGC CTGCGAGATC
3241 TGGCCGTGGC TGTGGAACCA GTCGTCTTCT CCCGAATGGA GACCAAGCTC ATCACGTGGG
3301 GGGCAGATAC CGCCGCGTGC GGTGACATCA TCAACGGCTT GCCCGTCTCT GCCCGTAGGG

3361 GCCAGGAGAT ACTGCTTGGG CCAGCCGACG GAATGGTCTC CAAGGGGTGG AGGTTGCTGG
3421 CGCCCATCAC GGCGTACGCC CAGCAGACGA GAGGCCTCCT AGGGTGTATA ATCACCAGCC
3481 TGACTGGCCG GGACAAAAAC CAAGTGGAGG GTGAGGTCCA GATCGTGTCA ACTGCTACCC
3541 AAACCTTCCT GGCAACGTGC ATCAATGGGG TATGCTGGAC TGTCTACCAC GGGGCCGGAA
3601 CGAGGACCAT CGCATCACCC AAGGGTCCTG TCATCCAGAT GTATACCAAT GTGGACCAAG
3661 ACCTTGTGGG CTGGCCCGCT CCTCAAGGTT CCCGCTCATT GACACCCTGC ACCTGCGGCT
3721 CCTCGGACCT TTACCTGGTC ACGAGGCACG CCGATGTCAT T**CCCGTGCGC** **CG**GCGAGGTG
3781 ATAGCAGGGG TAGCCTGCTT TCGCCCCGGC CCATTTCCTA CTTGAAAGGC TCCTCGGGGG
3841 GTCCGCTGTT GTGCCCCGCG GGACACGCCG TGGGCCTATT CAGGGCCGCG GTGTGCACCC
3901 GTGGAGTGGC TAAGGCGGTG GACTTTATCC CTGTGGAGAA CCTAGAGACA ACCATGAGAT
3961 CCCCGGTGTT CACGGACAAC TCCTCTCCAC CAGCAGTGCC CCAGAGCTTC CAGGTGGCCC
4021 ACCTGCATGC TCCCACCGGC AGCGGTAAGA GCACCAAGGT CCCGGCTGCG TACGCAGCCC
4081 AGGGCTACAA GGTGTTGGTG CTCAACCCCT CTGTTGCTGC AACGCTGGGC TTTGGTGCTT
4141 ACATGTCCAA GGCCCATGGG GTTGATCCTA ATATCAGGAC CGGGGTGAGA ACAATTACCA
4201 CTGGCAGCCC CATCACGTAC TCCACCTACG GCAAGTTC**CT** **TGCCGACG**GC GGGTGCTCAG
4261 GAGGTGCTTA TGACATAATA ATTTGTGACG AGTGCCACTC CACGGATGCC ACATCCATCT
4321 TGGGCATCGG CACTGTCCTT GACCAAGCAG AGACTGCGGG GGCGAGACTG GTTGTGCTCG
4381 CCACTGCTAC CCCTCCGGGC TCCGTCACTG TGTCCCATCC TAACATCGAG GAGGTTGCTC
4441 TGTCCACCAC CGGAGAGATC CCTTTTTACG GCAAGGCTAT CCCCCTCGAG GTGATCAAGG
4501 GGGGAAGACA TCTCATCTTC TGCCACTCAA AGAAGAAGTG CGACGAGCTC GCCGCGAAGC
4561 TGGTCGCATT GGGCATCAAT GCCGTGGCCT ACTACCGCGG TCTTGACGTG TCTGTCATCC
4621 CGACCAGCGG CGATGTTGTC GTCGTGTCGA CCGATGCTCT CATGACTGGC TTTACCGGCG
4681 ACTTCGACTC TGTGATAGAC TGCAACACGT GTGTCACTCA GACAGTCGAT TTCAGCCTTG
4741 ACCCTACCTT TACCATTGAG ACAACCACGC TCCCCCAGGA TGCTGTCTCC AGGACTCAAC
4801 GCCGGGGCAG GACTGGCAGG GGGAAGCCAG GCATCTACAG ATTTGTGGCA CCGGGGGAGC
4861 GCCCCTCCGG CATGTTCGAC TCGTCCGTCC TCTGTGAGTG CTATGACGCG GGCTGTGCTT
4921 GGTATGAGCT CACGCCCGCC GAGACTACAG TTAGGCTACG AGCGTACATG AACACCCCGG
4981 GGCTTCCCGT GTGCCAGGAC CATCTTGAAT TTTGGGAGGG CGTCTTTACG GGCCTCACTC
5041 ATATAGATGC CCACTTTCTA TCCCAGACAA AGCAGAGTGG GGAGAACTTT CCTTACCTGG
5101 TAGCGTACCA AGCCACCGTG TGCGCTAGGG CTCAAGCCCC TCCCCCATCG TGGGACCAGA
5161 TGTGGAAGTG TTTGATCCGC CTTAAACCCA CCCTCCATGG GCCAACACCC CTGCTATACA
5221 GACTGGGCGC TGTTCAGAAT GAAGTCACCC TGACGCACCC AATCACCAAA TACATCATGA
5281 CATGCATGTC GGCCGACCTG GAGGTCGTCA CGAGCACCTG GGTGCTCGTT GGCGGCGTCC
5341 TGGCTGCTCT GGCCGCGTAT TGCCTGTCAA CAGGCTGCGT GGTCATAGTG GGCAGGATTG
5401 TCTTGTCCGG GAAGCCGGCA ATTATACCTG ACAGGGAGGT TCTCTACCAG GAGTTCGATG
5461 AGATGGAAGA GTGCTCTCAG CACTTACCGT ACATCGAGCA AGGGATGATG CTCGCTGAGC
5521 AGTTCAAGCA GAAGGCCCTC GGCCTCCTGC AGACCGCGTC CCGCCAAGCA GAGGTTATCA
5581 CCCCTGCTGT CCAGACCAAC TGGCAGAAAC TCGAGGTCTT CTGGGCGAAG CACATGTGGA
5641 ATTTCATCAG TGGGATACAA TACTTGGCGG GCCTGTCAAC GCTGCCTGGT AACCCCGCCA
5701 TTGCTTCATT GATGGCTTTT ACAGCTGCCG TCACCAGCCC ACTAACCACT GGCCAAACCC
5761 TCCTCTTCAA CATATTGGGG GGGTGGGTGG CTGCCCAGCT CGCCGCCCCC GGTGCCGCTA
5821 CCGCCTTTGT GGGCGCTGGC TTAGCTGGCG CCGCCATCGG CAGCGTTGGA CTGGGGAAGG
5881 TCCTCGTGGA CATTCTTGCA GGGTATGGCG CGGGCGTGGC GGGAGCTCTT GTAGCATTCA
5941 AGATCATGAG CGGTGAGGTC CCCTCCACGG AGGACCTGGT CAATCTGCTG CCCGCCATCC
6001 TCTCGCCTGG AG**CCCTTGTA** **GTCG**GTGTGG TCTGCGCAG**C** **AATACTGCGC** **CG**GCACGTTG
6061 GCCCGGGCGA GGGGGCAGTG CAATGGATGA ACCGGCTAAT AGCCTTCGCC TCCCGGGGGA
6121 ACCATGTTTC CCCCACGCAC TACGTGCCGG AGAGCGATGC AGCCGCCCGC GTCACTGCCA
6181 TACTCAGCAG CCTCACTGTA ACCCAGCTCC TGAGGCGACT GCATCAGTGG ATAAGCTCGG
6241 AGTGTACCAC TCCATGCTCC GGTTCCTGGC TAAGGGACAT CTGGGACTGG ATATGCGAGG
6301 TGCTGAGCGA CTTTAAGACC TGGCTGAAAG CCAAGCTCAT GCCACAACTG CCTGGGATTC
6361 CCTTTGTGTC CTGCCAGCGC GGGTATAGGG GGGTCTGGCG AGGAGACGGC ATTATGCACA
6421 CTCGCTGCCA CTGTGGAGCT GAGATCACTG GACATGTCAA AAACGGGACG ATGAGGATCG
6481 TCGGTCCTAG GACCTGCAGG AACATGTGGA GTGGGACGTT CCCCATTAAC GCCTACACCA
6541 CGGGCCCCTG TACTCCCCTT CCTGCGCCGA ACTATAAGTT CGCGCTGTGG AGGGTGTCTG
6601 CAGAGGAATA CGTGGAGATA AGGCGGGTGG GGGACTTCCA CTACGTATCG GGTATGACTA
6661 CTGACAATCT TAAATGCCCG TGCCAGATCC CATCGCCCGA ATTTTTCACA GAATTGGACG
6721 GGGTGCGCCT ACATAGGTTT GCGCCCCCTT GCAAGCCCTT GCTGCGGGAG GAGGTATCAT
6781 TCAGAGTAGG ACTCCACGAG TACCCGGTGG GGTCGCAATT ACCTTGCGAG CCCGAACCGG
6841 ACGTAGCCGT GTTGACGTCC ATGCTCACTG ATCCCTCCCA TATAACAGCA GAGGCGGCCG
6901 GGAGAAGGTT GGCGAGAGGG TCACCCCCTT CTATGGCCAG CTCCTCGGCC AGCCAGCTGT
6961 CCGCTCCATC TCTCAAGGCA ACTTGCACCG CCAACCATGA CTCCCCTGAC GCCGAGCTCA

7021 TAGAGGCTAA CCTCCTGTGG AGGCAGGAGA TGGGCGGCAA CATCACCAGG GTTGAGTCAG
7081 AGAACAAAGT GGTGATTCTG GACTCCTTCG ATCCGCTTGT GGCAGAGGAG GATGAGCGGG
7141 AGGTCTCCGT ACCCGCAGAA ATTCTGCGGA AGTCTCGGAG ATTCGCCCGG GCCCTGCCCG
7201 TTTGGGCGCG GCCGGACTAC AACCCCCCGC TAGTAGAGAC GTGGAAAAAG CCTGACTACG
7261 AACCACCTGT GGTCCATGGC TGCCCGCTAC CACCTCCACG GTCCCCTCCT GTGCCTCCGC
7321 CTCGGAAAAA GCGTACGGTG GTCCTCACCG AATCAACCCT ATCTACTGCC TTGGCCGAGC
7381 TTGCCACCAA AAGTTTTGGC AGCTCCTCAA CTTCCGGCAT TACGGGCGAC AATACGACAA
7441 CATCCTCTGA GCCCGCCCCT TCTGGCTGCC CCCCCGACTC CGACGTTGAG TCCTATTCTT
7501 CCATGCCCCC CCTGGAGGGG GAGCCTGGGG ATCCGGATCT CAGCGACGGG TCATGGTCGA
7561 CGGTCAGTAG TGGGGCCGAC ACGGAAGATG TCGTGTGCTG CTCAATGTCT TATTC**CTGGA**
7621 **CAGGCG**CACT CGTCACCCCG TGCGCTGCGG AAGAACAAAA ACTGCCCATC AACGCACTGA
7681 GCAACTCGTT GCTACGCCAT CACAATCTGG TGTATTCCAC CACTTCACGC AGTGCTTGCC
7741 AAAGGCAGAA GAAAGTCACA TTTGACAGAC TGCAAGTTCT GGACAGCCAT TACCAGGACG
7801 TGCTCAAGGA GGTCAAAGCA GCGGCGTCAA AAGTGAAGGC TAACTTGCTA TCCGTAGAGG
7861 AAGCTTGCAG CCTGACGCCC CCACATTCAG CCAAATCCAA GTTTGGCTAT GGGG**CAAAAG**
7921 **ACG**TCCGTTG CCATGCCAGA AAGGCCGTAG CCCACATCAA CTCCGTGTGG AAAGACCTTC
7981 TGGAAGACAG TGTAACACCA ATAGACACTA CCATCATGGC CAAGAACGAG GTTTTCTGCG
8041 TTCAGCCTGA GAAGGGGGGT CGTAAGCCAG CTCGTCTCAT CGTGTTCCCC GACCTGGGCG
8101 TGCGCGTGTG CGAGAAGATG G**CCCTGTACG** **ACG**TGGTTAG CAAGCTCCCC CTGGCCGTGA
8161 TGGGAAGCTC CTACGGATTC CAATACTCAC CAGGACAGCG GGTTGAATTC CTCGTGCAAG
8221 CGTGGAAGTC CAAGAAGACC CCGATGGGGT TCTCGTATGA TACCCGCTGT TTTGACTCCA
8281 CAGTCACTGA GAGCGACATC CGTACGGAGG AGGCAATTTA CCAATGTTGT GACCTGGACC
8341 CCCAAGCCCG CGTGGCCATC AAGTCCCTCA CTGAGAGGCT TTATGTTGGG GGCCCTCTTA
8401 CCAATTCAAG GGGGGAAAAC TGCGGCTAC**C** **GCAGGTGCCG** CGCGAGCGGC GTACTGACAA
8461 CTAGCTGTGG TAACACCCTC ACTTGCTACA TCAAGGCCCG GGCAGCCTGT CGAGCCGCAG
8521 GGCTCCAGGA CTGCACCATG CTCGTGTGTG GCGACGACTT AGTCGTTATC TGTGAAAGTG
8581 CGGGGGTCCA GGAGGACGCG GCGAGCCTGA GAGCCTTCAC GGAGGCTATG ACCAGGTACT
8641 CCGCCCCCCC CGGGGACCCC CCACAACCAG AATACGACTT GGAGCTTATA ACATCATGCT
8701 CCTCCAACGT GTCAGTCGCC CACGACGGCG CTGGAAAGAG GGTCTACTAC CTTACCCGTG
8761 ACCCTACAAC CCCCCTCGCG AGAGCCGCGT GGGAGACAGC AAGACACACT CCAGTCAATT
8821 CCTGGCTAGG CAACATAATC ATGTTTGCCC CCACACTGTG GGCGAGGATG ATACTGATGA
8881 CCCATTTCTT TAGCGTCCTC ATAGCCAGGG ATCAGCTTGA ACAGGCTCTT AACTGTGAGA
8941 TCTACGGAGC CTGCTACTCC ATAGAACCAC TGGATCTACC TCCAATCATT CAAAGACTCC
9001 ATGGCCTCAG CGCATTTTCA CTCCACAGTT ACTCTCCAGG TGAAATCAAT AGGGTGGCCG
9061 CATGCCTCAG AAAACTTGGG GTCCCGCCCT TGCGAGCTTG GAGACACCGG GCCCGGAGCG
9121 TCCGCGCTAG GCTTCTGTCC AGAGGAGGCA GGGCTGCCAT ATGTGGCAAG TACCTCTTCA
9181 ACTGGGCAGT AAGAACAAAG CTCAAACTCA CTCCAATAGC GGCCGCTGGC CGGCTGGACT
9241 TGTCCGGTTG GTTCACGGCT GGCTACAGCG GGGGAGACAT TTATCACAGC GTGTCTCATG
9301 CCCGGCCCCG CTGGTTCTGG TTTTGCCTAC TCCTGCTCGC TGCAGGGGTA GGCATCTACC
9361 TCCTCCCCAA CCGATGAAGG TTGGGGTAAA CACTCCGGCC TCTTAGGCCA TTTCCTGTTT
9421 TTTTTTTTTT TTTTTTTTTT TTTTTTTTTT TTTTTTTTTT TTTTTTTTCT TTTTTTTTTT
9481 TTTTTTCCTT TTTTTTTTTT TTTTTTTTCT TTCCTTCTTT TTTCCTTTCT TTTCCTTCCT
9541 TCTTTAATGG TGGCTCCATC TTAGCCCTAG TCACGGCTAG CTGTGAAAGG TCCGTGAGCC
9601 GCATGACTGC AGAGAGTGCT GATACTGGCC TCTCTGCAGA TCATGT

Figure S3. Analysis of conservation of ZAP-binding motifs in the HCV reference sequence (NC 004102): Sequences marked in red are ZAP-binding motifs, and the bold sequences are the ZAP-binding motifs that are conserved across at least 75% of the 2616 sequences analysed.
